# Teacher Efficacy, Work Engagement, and Social Support Among Chinese Special Education School Teachers

**DOI:** 10.3389/fpsyg.2018.00648

**Published:** 2018-05-07

**Authors:** Lu Minghui, Hao Lei, Chen Xiaomeng, Miloň Potměšilc

**Affiliations:** ^1^Special Education Department, School of Education, Guangzhou University, Guangzhou, China; ^2^Institute of Curriculum and Instruction, East China Normal University, Shanghai, China; ^3^School of Education, South China Normal University, Guangzhou, China; ^4^Institute of Special Education Studies, Faculty of Education, Palacký University, Olomouc, Czechia

**Keywords:** Chinese special education school teachers, social support, teacher efficacy, work engagement

## Abstract

This paper investigates the relationship between teacher efficacy and socio-demographic factors, work engagement, and social support among Chinese special education school teachers. The sample comprised 1,027 special education school teachers in mainland China. The Teachers’ Sense of Efficacy Scale, the Multi-Dimensional Scale of Perceived Social Support, and the Utrecht Work Engagement Scale were used for data collection. Correlation analysis revealed that social support, work engagement, and teacher efficacy were significantly correlated with each other. Additionally, gender, years of experience, and monthly salary were significant predictors of teacher efficacy. Furthermore, structural equation modeling analysis showed that social support exerted its indirect effect on teacher efficacy through the mediation of work engagement. The findings of this study provide a new perspective on the complex association between social support and teacher efficacy. The explanations and limitations of these findings are discussed.

## Introduction

In recent years, special education in China has changed in positive ways as a result of the government’s education reforms. These changes include emphasizing the importance of teachers. The quality of teachers has been recognized as one of the most important factors in developing and improving special education in China (Wang and Mu, 2014). Presently, the vast majority of students with special education needs are sent to special schools, and teachers in these schools face severe stress because of a paucity of practical training, high student-faculty ratios, insufficient facilities, and a shortage of professional skills (Lai et al., 2016; Zee et al., 2016). In order to teach effectively, teachers must compensate for this deficient teaching environment. The literature also shows that burnout occurs frequently among special education teachers (Sariçam and Sakiz, 2014). These phenomena indicate that teachers’ characteristics merit further investigation so that specific interventions to improve their work may be developed. Traditionally, teachers’ characteristics have been measured on the basis of subject knowledge, certification, and experience. However, in the past decade, research has also acknowledged the importance of teachers’ attitudes and beliefs about their teaching to student progress and achievement (Ekstam et al., 2017). Therefore, it is also important to investigate factors, such as social context, job satisfaction, and work engagement, that contribute to shaping these beliefs. In China, it is particularly essential to assess such factors for special education school teachers comprehensively because of the high levels of cultural stigma that children with disabilities and their teachers face. However, there has been limited research on these issues in China.

### Teacher Efficacy

The development of teacher efficacy is in part due to two similar learning theories: Rotter’s social learning theory (1966) and Bandura’s social cognitive theory (1977) (Tschannen-Moran and Hoy, 2001; McCarty, 2013). Teacher efficacy is defined as a teacher’s beliefs or perceptions about his or her ability to teach students with different kinds of needs and to bring about desired changes in students’ achievement (Tschannen-Moran et al., 1998). Studies have indicated that teacher efficacy is one of the most pervasive factors that potentially distinguishes teachers who teach effectively from those who usually struggle in teaching (Tschannen-Moran and Hoy, 2001). When examining teacher efficacy, researchers discovered that one teacher may have differing perceptions of efficacy when teaching different students or in varying situations. Teaching in a special education setting differs greatly from teaching students in a general education setting (McCarty, 2013). Special education school teachers are directly responsible for providing appropriate educational interventions for students who have speech disorders, cognitive deficiencies, attention deficit/hyperactivity disorder, or other behavioral problems (Skuller, 2011). The effectiveness of teachers’ teaching ability, their persistence in the face of frustration, their instructional behaviors, and the performance of their students are influenced significantly by teacher efficacy (Pacheco and García, 2012; Dixon et al., 2014; Scherer et al., 2016). Therefore, it is important to perform a specific examination of special education school teachers’ sense of teacher efficacy.

### Social Support

Social support is defined as the provision of physical, emotional, informational, and instrumental assistance that an individual perceives from his or her social networks (Cobb, 1976; Lu et al., 2015). Social support encompasses a multitude of social interactions with one’s spouse, extended family, friends, and others (Siklos and Kerns, 2006). Previous studies have found that teachers of special education in China are more likely to experience a sense of social isolation and prejudice than those in developed counties because of a general lack of public awareness about the meaning and significance of educating children with special needs (Mcloughlin et al., 2005; Zheng and Zheng, 2015). From this perspective, social support is of great significance in the process of fostering Chinese special education school teachers.

Studies have suggested that social support is an essential factor in teachers’ efficacy and psychological state (Wallace et al., 2001; Shen, 2009) and that their psychological well-being can be enhanced by investing in their social context (Kruger, 1997; Field and Buitendach, 2012). Studies have also indicated that social support, both informal (e.g., from friends and relatives) and formal (e.g., from organizations), appears necessary for teachers to improve the quality of their teaching (Skaalvik and Skaalvik, 2009; Fiorilli et al., 2015). In special education, perceived social support might contribute to their openness to new experiences, feelings of burnout, and beliefs about teaching activities (Combee, 2014).

### Work Engagement

Work engagement is characterized by vigor, dedication, and absorption. It represents a positive and psychologically fulfilling state of mind (Bakker et al., 2008). The concept has become a core indicator that reflects the quality of teachers’ occupational lives by accounting for significant variation in the prediction of their occupational and organizational outcomes, such as teaching performance, problem solving, organizational commitment, and job satisfaction (Hakanen et al., 2006; Field and Buitendach, 2012). Furthermore, research has shown that work engagement was significantly and positively correlated with teacher efficacy (Hoigaard et al., 2012).

Work engagement may mediate between social support and teacher efficacy. According to the job demands-resources model, resources such as social support will help teachers cope with the emotional demands of teaching and will affect teachers’ engagement (Hultell and Gustavsson, 2011). Previous studies also showed that changes in engagement levels are strongly tied to changes in senses of efficacy (Bresó et al., 2011). Although some ideas about how work engagement mediates teacher efficacy have been proposed in the research literature, few scholars have explored this key topic with regard to special education teachers.

### The Current Study

At present, Chinese special education teachers suffer from prejudice and have to juggle numerous difficult tasks in addition to students’ unique needs in their daily practice. However, to date, little research has been conducted about the relations among teacher efficacy, social support, and work engagement for special education.

The present investigation aims to contribute to our understanding of teacher efficacy and the related factors of socio-demographic factors, social support and work engagement among teachers at Chinese special education schools. Given the preceding rationale and the available literature, this study hypothesizes that teacher efficacy is associated with socio-demographic factors, social support, and work engagement. Specifically, the relationship between social support and teacher efficacy is mediated by work engagement. We hope that our study can provide evidence-based information that may prompt policy makers and professionals to provide better support for teachers at special education schools and assist them in adapting to their role, which would improve the quality of their teaching.

## Materials and Methods

### Participants

The participants in this study comprised a convenience sample of 1027 special education teachers from special education schools in China. The 1027 participants included 232 males (24.6%) and 775 females (75.4%). The mean age of the sample was 36.22 years (*SD* = 7.56 years). The teachers’ average experience in terms of years was 11.65 (*SD* = 6.31). The demographic characteristics of the participants are further illustrated in **Table 1**.

**Table 1:** Socio-demographic characteristics of the sample.

Demographic variables	Female	Male	Total
Number	775 (75.4%)	252 (24.6%)	1027 (100%)
Age (*M* ± *SD*)	35.43 ± 7.66	38.21 ± 7.39	36.22 ± 7.56
Years’ experience (*M* ± *SD*)	11.09 ± 7.80	13.81 ± 7.99	11.65 ± 6.31
Monthly salary			
≤4000 RMB ($634.92)	287	88	375 (36.6%)
4000–6000 RMB ($634.92–$952.38)	268	96	364 (35.4%)
6000–8000 RMB ($952.38–$1269.84)	128	54	182 (17.7%)
≥8000 RMB ($1269.84)	92	14	106 (10.3%)
Whether satisfied with salary			
Satisfied with salary	508	121	629 (61.3%)
Not satisfied with salary	267	131	398 (38.7%)

*1 US dollar is equal to approximately 6.30 RMB.*

### Procedure

The cross-sectional survey was conducted in mainland China. Teachers were selected from special education schools. A detailed description of the study, as well as the intended use of the results, was provided to each teacher. Participants were invited to complete a package of questionnaires, including a socio-demographic information questionnaire, the Teachers’ Sense of Efficacy Scale (TSES), the Multi-Dimensional Scale of Perceived Social Support (MSPSS), and the Utrecht Work Engagement Scale (UWES). The questionnaire data were kept confidential in order to protect the anonymity of teachers. All the participating teachers were volunteers and did not receive any monetary compensation. All participants provided their written informed consent to participate in this study and the study was reviewed and approved by Human Research Ethics Committee for Non-Clinical Faculties (ethics committee from the School of Education, Guangzhou University) before the study began.

### Measures

#### Brief Demographic Questionnaire and Familial Profiles

Questions were asked about the age, gender, education level, and marital status, as well as years of experience and monthly salary.

#### Teachers’ Sense of Efficacy Scale (TSES)

The TSES was originally developed by Tschannen-Moran and Hoy (2001). The TSES is a reliable and valid instrument that measures a teacher’s general sense of efficacy. It contains 24 items on a nine-point Likert scale and three subscale measures, including instructional strategies, classroom management, and student engagement (1 = nothing, 9 = a great deal). The total scale ranges from 24 to 216, with higher scores indicating higher teacher efficacy. “Student engagement” refers to a teacher’s behavior to ensure that students are actively engaged in the learning process, and “classroom management” means a teacher’s ability to direct the flow of a class toward a set of learning objectives. Finally, “instructional strategies” encompass a teacher’s competence in employing proper pedagogy to ensure student learning. In the current study, the Cronbach’s alpha coefficient of TSES was 0.86.

#### Multi-Dimensional Scale of Perceived Social Support (MSPSS)

The MSPSS was developed by Zimet et al. (1988). It consists of 12 items rated on a seven-point Likert scale and three subscale measures, including family support, friend support and other support (1 = very strongly disagree, 7 = very strongly agree). The total score ranges from 12 to 84, with higher scores indicating more perceived social support. The Chinese version of the MSPSS has good reliability and validity (Zhao et al., 2014; Tu and Yang, 2016). In the current study, the Cronbach’s alpha coefficient for the MSPSS was 0.83.

#### Utrecht Work Engagement Scale (UWES)

The Utrecht Work Engagement Scale (UWES) was developed by Schaufeli et al. (2002). It contains 17 items on a seven-point Likert scale and three subscale measures, including vigor, dedication, and absorption (0 = never, 6 = always). Vigor is characterized by high levels of energy, resilience, and persistence in the face of obstacles and difficulties. Dedication refers to a sense of enthusiasm and inspiration. Absorption means full concentration on one’s teaching tasks. The total score ranges from 0 to 102, with high scores indicating greater levels of work engagement. The Chinese version of UWES has good reliability and validity (Li et al., 2015). In the current study, the Cronbach’s alpha coefficient for the UWES was 0.87.

### Statistical Analysis

All statistical analyses were performed using SPSS 21.0 and AMOS 21.0. The statistical descriptions comprised the mean, standard deviation, frequencies, and percentages. Pearson’s correlation analysis was calculated for social support, work engagement, and teacher efficacy. Hierarchical multiple regression analysis was performed to determine the associations of teacher efficacy, social support, and work engagement when controlling for socio-demographic factors. In the regression procedure, the total score for teacher efficacy was taken as the dependent variable. Independent variables were entered in the following order: socio-demographic factors in step 1; social support and work engagement subscales in step 2. All statistical tests were evaluated at the *p* < 0.05 significance level and constituted two-tailed tests. The SEM procedure was employed to test how work engagement mediates the relationship between social support and teacher efficacy. Consistently with the recommendation of Zhonglin et al. (2004), the goodness of fit of the structural model was evaluated using the following indices: RMSEA (best if below 0.06), SRMR (best if below 0.08), NFI, CFI, TLI, IFI, and RFI (best if above 0.95). The research variables are further illustrated in **Table 2**.

**Table 2:** Descriptive statistics and correlations between the measured variables.

	*M*	*SD*	1	2	3	4	5	6	7	8	9
(1) FAS	22.11	4.27	1								
(2) FRS	21.20	4.10	0.555^∗∗^	1							
(3) OS	20.66	4.13	0.516^∗∗^	0.636^∗∗^	1						
(4) VI	20.03	8.69	0.192^∗∗^	0.210^∗∗^	0.278^∗∗^	1					
(5) DE	17.18	8.03	0.181^∗∗^	0.243^∗∗^	0.274^∗∗^	0.758^∗∗^	1				
(6) AB	18.95	6.37	0.219^∗∗^	0.270^∗∗^	0.318^∗∗^	0.710^∗∗^	0.741^∗∗^	1			
(7) SE	48.03	9.44	0.375^∗∗^	0.329^∗∗^	0.330^∗∗^	0.251^∗∗^	0.208^∗∗^	0.236^∗∗^	1		
(8) IS	50.43	8.69	0.389^∗∗^	0.335^∗∗^	0.294^∗∗^	0.224^∗∗^	0.187^∗∗^	0.233^∗∗^	0.731^∗∗^	1	
(9) CM	53.21	9.19	0.364^∗∗^	0.295^∗∗^	0.237^∗∗^	0.219^∗∗^	0.190^∗∗^	0.179^∗∗^	0.723^∗∗^	0.698^∗∗^	1

*^∗∗^p < 0.01; FAS, family support; FRS, friend support; OS, other support; VI, vigor; DE, dedication; AB, absorption; SE, student engagement; IS, instructional strategies; CM, classroom management.*

## Results

### Descriptive Analyses

The means and standard deviations of the MSPSS, UWES, and TSES are shown in **Table 2**. Pearson correlations showed that the variables were significantly correlated. Pearson product-moment correlation coefficients were all positive and significant at the *p* < 0.01 level.

### Multiple-Regression Analysis of Teacher Efficacy

**Table 3** summarizes the results of hierarchical multiple-regression analysis. In step 1 of the analysis, socio-demographic factors accounted for 5.9% (*R*^2^ = 0.059, *p* < 0.01) of the variation in teacher efficacy, while the gender of the teachers, their years of experience, and their monthly salaries were statistically significant. The introduction of social support and work engagement subscales in step 2 accounted for 19.7% (*R*^2^ change = 0.197, *p* < 0.01) of the variation in teacher efficacy. Steps 1 and 2 combined accounted for 25.6% (*R*^2^ = 0.256, *p* < 0.01) of the variance in teacher efficacy, while the gender of the teachers, their years of experience, support from family, support from friends, and the characteristic of vigor were statistically significant.

**Table 3:** Hierarchical multiple regression results predicting teacher efficacy.

Predictor	Step 1		Step 2	
	β	*t*	β	*t*
Gender of teachers				
Female	0.091	2.356^∗^	0.067	2.273^∗^
Years’ of experience	0.197	6.536^∗∗^	0.134	4.548^∗∗^
Monthly salary				
4000–6000 RMB	0.117	2.673^∗^	0.054	1.099
6000–8000 RMB	0.165	2.972^∗^	0.104	2.677^∗^
≥8000 RMB	0.281	3.185^∗∗^	0.189	2.915^∗^
Family support			0.246	6.730^∗∗^
Friend support			0.168	4.117^∗∗^
Other support			0.007	0.171
Vigor			0.301	3.833^∗∗^
Dedication			0.066	1.018
Absorption			0.088	1.195
*R*^2^	0.059		0.256	
*F*	22.263^∗∗^		40.507^∗∗^	
ΔR^2^	0.059		0.197	
ΔF	22.263^∗∗^		15.379^∗∗^	

*^∗^p < 0.05, ^∗∗^p < 0.01.*

### The Mediating Effect of Work Engagement on the Relationship Between Social Support and Teacher Efficacy

**FIGURE 1:**
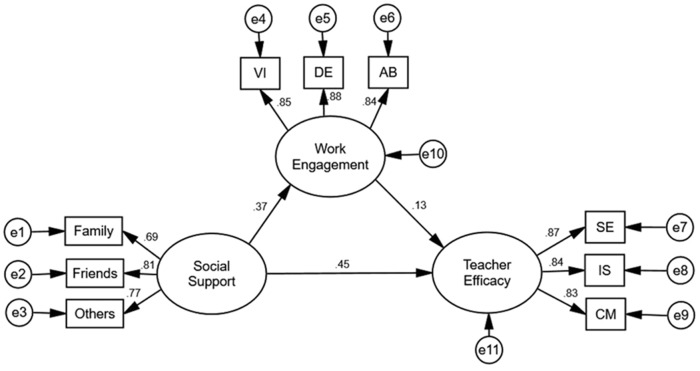
The partial mediation model (*N* = 1027). Factor loadings are standardized. The mediation model shows the independent variable (social support), mediator (work engagement), and dependent variable (teacher efficacy). Rectangles represent observed variables. Circles represent latent variables. VI, vigor; DE, dedication; AB, absorption; SE, student engagement; IS, instructional strategies; CM, classroom management.

The SEM procedure was employed to test the proposed structural relationships among social support, work engagement, and teacher efficacy. The measurement model consisted of three latent factors (social support, work engagement, teacher efficacy) and nine observed variables. The mediational model, including both indirect pathways and direct pathways, was tested using chi-square differences. As shown in **Table 4**, the results demonstrated that the fit indices of the partial mediation model (χ^2^ = 96.862, df = 24, *p* < 0.001, RMSEA = 0.054, 90% CI = 0.043–0.066, SRMR = 0.033, NFI = 0.980, TLI = 0.978, CFI = 0.985, IFI = 0.985, RFI = 0.970) were good and met the psychometric standard. These results suggested that work engagement may play a partial mediating role in the relationship between social support and teacher efficacy. The standardized path coefficients for the partial mediation model are displayed in **Figure 1**.

**Table 4:** Fit indices of the partial mediation model.

χ^2^	df	χ^2^/df	RMSEA	SRMR	NFI	TLI	CFI	IFI	RFI
96.862	24	4.036	0.054	0.033	0.980	0.978	0.985	0.985	0.970

*N = 1027.*

The Bootstrap test was used to examine the significance of the mediating effects of work engagement on the relationship between social support and teacher efficacy. We generated 1000 bootstrapping samples from the original data set by random sampling. The results showed that the indirect effect was significant at the 0.01 level (indirect effect = 0.046, *p* < 0.01, 95% CI = 0.022, 0.073), which accounted for 9.3% of the total effects (0.497).

These findings indicate that work engagement does indeed play a partial mediating role in the relationship between social support and teacher efficacy.

## Discussion

In 2006, a national survey of China indicated that nearly 84 million people, or 6.34% of its population, have disabilities (Zhang and Spencer, 2015). However, education for people with disabilities has not received adequate attention until recent years, and the quality of teaching is still a universal challenge for most special education schools in China. Among the multiple indicators that reflect the quality of teaching, teacher efficacy has received widespread attention. However, little research has examined teacher efficacy in Chinese special education school teachers.

The current study investigated the prediction effect of socio-demographic factors, social support, and work engagement on teacher efficacy in Chinese special education schools. The following sections discuss the relationship of these selected variables.

### Associations Between Teacher Efficacy and Socio-Demographic Factors

As shown in the present study, socio-demographic factors associated with teacher efficacy included gender, years of experience, and monthly salary. The present study found that female teachers outperformed male teachers in terms of teacher efficacy, which is consistent with a previous study conducted in Taiwan (Lee, 2000). According to the literature and practical situations, this result may reflect the existence of stereotype that a female teacher may be thought of as more suitable for work in special education when the Chinese cultural background is considered.

Monthly salary is a significant predictor of teacher efficacy. A previous study has suggested that there is a connection between teacher efficacy and salary (McCarty, 2013), and this study is consistent with the finding. In addition to mental and emotional burdens, special education school teachers bear the financial responsibility for supporting their families. As shown in the present study, approximately 61.3% of teachers are not satisfied with their monthly salaries. As a secondary intervention, financial support for teachers may contribute to a greater sense of well-being and thus better equip them to care for children with special needs.

Years of experience are another significant predictor of teacher efficacy; this finding is consistent with those of previous studies (Woolfolk and Hoy, 1990; Lamorey and Wilcox, 2005). As a result of the inadequate pre-service training in special education that Chinese teachers receive, on-the-job training and practice is the main way to improve teaching skills. This result suggests that, with more teaching experience, teachers may feel increasingly positive about their growing knowledge and skills, and therefore judge themselves as increasingly successful in special education.

### Associations Between Teacher Efficacy, Social Support, and Work Engagement

The present study confirmed the associations between teacher efficacy, social support, and work engagement. Furthermore, hierarchical multiple regression analysis showed that only the characteristic of vigor and the support of family and friends, rather than other kinds of support or the characteristics of dedication or absorption, were significantly associated with teacher efficacy when controlling for socio-demographic factors. It is likely that different aspects of social support and work engagement weigh differently in their associations with teacher efficacy. Specifically, the support of family and friends and the characteristic of vigor play the most important roles.

### The Mediating Role of Work Engagement

Although measures of social support and teacher efficacy are reliably correlated, the degree to which intervening variables mediate their relationship is not clear. This study provides evidence for the mediating role of work engagement in the relation between social support and teacher efficacy. Given the cross-sectional nature of the study, the results are open to multiple interpretations. One possible interpretation is that social support fosters work engagement; therefore, the internal psychological significance of social support actually promotes teacher efficacy. A second interpretation of the mediational model indicates that work engagement may prompt special education teachers to mobilize their social support resources. In particular, one’s level of work engagement may encourage one to seek out support, as well as to utilize that support effectively. The way in which social support is used, in turn, may contribute to teacher efficacy. Thus, work engagement is transformed in some way by the properties of the teacher.

### Practical Implications

Studies have shown that improvements in teacher efficacy can lead to enhanced job satisfaction and feelings of competence and can reduce the risk of burnout (Dixon et al., 2014; Sariçam and Sakiz, 2014). The hypothesis of the current study that work engagement serves as a mediator for the relationship between social support and teacher efficacy is supported by the results. In line with the expectations, the direct effect of social support on teacher efficacy was significant, that is, special education school teachers with high levels of social support were more likely to feel greater teacher efficacy. The mediating effect of work engagement on the relationships between social support and teacher efficacy was significant. In other words, social support indirectly affected teacher efficacy through work engagement. On a clinical level, to improve teacher efficacy, it is important to implement interventions that strengthen work engagement by fostering social support systems in the whole society. A previous study indicated that teachers’ engagement can be fostered through the satisfaction of basic psychological needs (Klassen et al., 2012). Thus, it is important to reduce the social, emotional, physical, and even financial burdens of teachers working in special education schools in China, and to provide productive feedback about effective teaching in order to foster work engagement and personal achievement among teachers in special education schools. Another important way to promote teacher efficacy is through mastery experiences. Professional development activities, such as training, tutoring, and observations, can have a positive impact on teacher efficacy (Haverback and Parault, 2008).

Most importantly, each country, while learning from the successful experiences of other countries, has to examine and improve its own system within its own unique cultural, economic, political, and policy contexts (Ellsworth and Zhang, 2007). While the understanding of special education is increasing among service providers in China, the general population commonly holds inaccurate views of children with disabilities and their teachers. On the basis of such realities, it is essential to make varied efforts to enhance social support more effectively. Social support can emerge from multiple sources, and should be considered in a wider ecological context beyond family, friends, and significant others. It is apparent that professional support systems need to be developed for teachers in special education schools, and that more public education campaigns are needed to raise awareness of special education and reduce any associated stigma.

## Conclusion

This study helps to improve understanding of social support, work engagement, and the socio-demographics factors that are associated with teacher efficacy among Chinese special education school teachers. In conclusion, teacher efficacy was associated with gender, years of experience, monthly salary, the support of family, the support of friends, and the characteristic of vigor. Work engagement mediates between social support and teacher efficacy.

## Limitations

The strengths of the present study lie first in the methodology used for the sampling and the large sample size. In addition, validated instruments enhance the accuracy of the findings and make cross-cultural comparisons possible.

Despite the above strengths, this study should be considered in light of several limitations. First, considering the distinct socioeconomic and cultural characteristics of China, our findings may not simply be generalized to other countries. Second, the data were collected through self-report questionnaires that largely depend on subjective observation, which may raise questions about accuracy. Third, other factors, such as social environments and teaching practice, may also affect teacher efficacy, which should be considered in future studies. Finally, it is also important to note that the questionnaires were obtained through the pencil and paper method and future research could obtain data in a virtual way through a professional survey application such as SurveyMonkey, which would speed up the data collection and avoid errors.

## Author Contributions

LM and LH provided the idea, designed this study and wrote the manuscript. CX contributed to data analysis and data collection. MP contributed to revised this manuscript.

## Conflict of Interest Statement

The authors declare that the research was conducted in the absence of any commercial or financial relationships that could be construed as a potential conflict of interest.
